# Electrochemical etching strategy for shaping monolithic 3D structures from 4H-SiC wafers

**DOI:** 10.1038/s41598-023-46110-2

**Published:** 2023-11-04

**Authors:** André Hochreiter, Fabian Groß, Morris-Niklas Möller, Michael Krieger, Heiko B. Weber

**Affiliations:** Department of Physics, Friedrich-Alexander-Universität Erlangen-Nürnberg (FAU), 91058 Erlangen, Germany

**Keywords:** Materials for devices, Electrical and electronic engineering

## Abstract

Silicon Carbide (SiC) is an outstanding material, not only for electronic applications, but also for projected functionalities in the realm of spin-based quantum technologies, nano-mechanical resonators and photonics-on-a-chip. For shaping 3D structures out of SiC wafers, predominantly dry-etching techniques are used. SiC is nearly inert with respect to wet etching, occasionally photoelectrochemical etching strategies have been applied. Here, we propose an electrochemical etching strategy that solely relies on defining etchable volumina by implantation of p-dopants. Together with the inertness of the n-doped regions, very sharp etching contrasts can be achieved. We present devices as different as monolithic cantilevers, disk-shaped optical resonators and membranes etched out of a single crystal wafer. The high quality of the resulting surfaces can even be enhanced by thermal treatment, with shape-stable devices up to and even beyond 1550°C. The versatility of our approach paves the way for new functionalities on SiC as high-performance multi-functional wafer platform.

## Introduction

Silicon Carbide (SiC), especially its polytype 4H-SiC, is an extraordinary material for integrating electronics^1^, photonics^2^, high-quality mechanics^3^ and quantum technologies on the very same chip^4,5^. Due to its technological breakthrough in power electronics, it is available as single crystalline high-quality wafers. When, further, optical and mechanical functionality is demanded, there is a need for highest-quality devices with three-dimensional geometries. As to optics, SiC provides the unusual opportunity of simultaneous χ^(2)^ and χ^(3)^ nonlinearities^6,7^. The current state-of-the-art photonics-on-a-chip is not integrated with traditional SiC fabrication techniques, but uses thin SiC-on-insulator technology^4,8,9^. As to mechanics-on-a-chip, SiC provides an outstanding intrinsic property: it has the lowest internal damping of all known materials^3,9,10^. Also here, device fabrication utilizes thin SiC layers on a sacrificial substrate. Given the extraordinary set of parameters of SiC, it is desirable to identify *monolithic* technologies for the preparation of optical and mechanical devices along with the electronic functionality. Compatibility with high-temperature protocols, for example epitaxial graphene growth^11^ or defect annealing^12^, would be beneficial.

**Figure 1:**
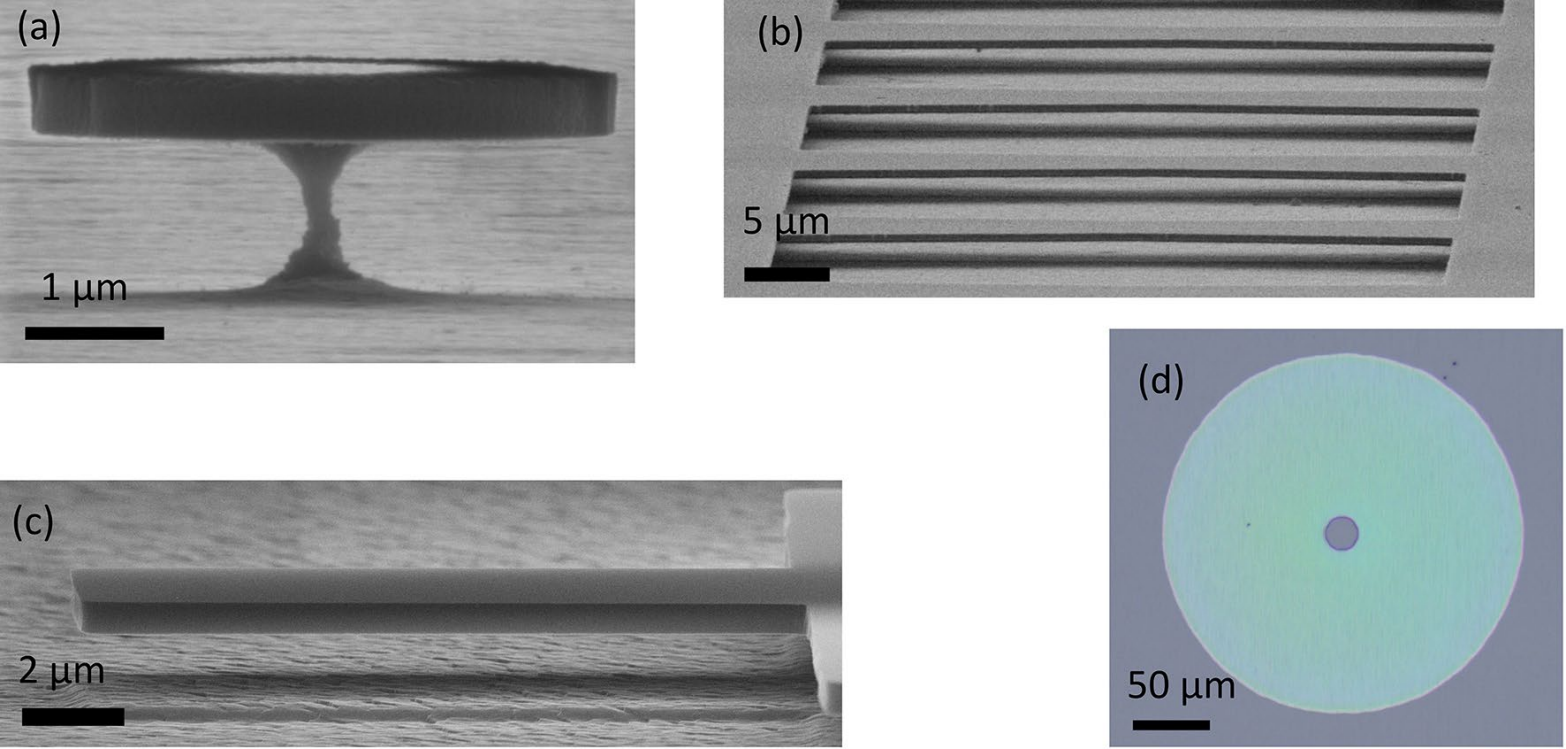
Monolithically etched 3D-devices from single-crystal 4H-SiC wafer. (**a**) disk-shape optical resonator, (**b**) doubly clamped mechanical resonator, (**c**) single clamped mechanical resonator, (**d**) free-standing circular membrane (central hole is required as etching access). (**a**–**c**) SEM micrographs, (**d**) optical micrograph.

For such applications, however, a technological prerequisite is an etching strategy that forms the desired 3D-structures monolithically out of the single-crystal wafer, while maintaining high-quality surfaces and low defect budgets. The commonly used gas etching strategies (ICP-RIE/RIE) are projective, and even ‘anisotropic’ gas etching has limited aspect ratios^13–15^. Further, they are prone to create surface-near point-defects. Therefore, a new process strategy is required. In an effort to increase the design space for advanced 3D geometries like cantilevers, disk-shaped resonators or membranes (cf. Fig. 1), including long-range lateral etching, we present a route based on implantation and subsequent electrochemical etching (ECE).

SiC is nearly inert and only few chemical reagents to resolve it under moderate conditions are known. Typically, both high temperatures and strong etchants are needed^16^. Here we choose positive charge carriers (holes, $$h^{ + } )$$ at the surface for the electrochemical attack. We opt for etching under alkaline conditions, the electrochemistry of which has extensively been studied by *van Dorp*^17–19^. In order to provide the required $$h^{ + }$$ on the surface, a vast majority of publications uses electron–hole creation by ultraviolet light including our own previous work^5,20–22^. This methodology is limited because of optical constraints, in particular it has poor vertical control^22^. Here we favor an electrochemical strategy where $$h^{ + }$$-concentrations are created by appropriate doping patterns. Additional control can be gained by electric potentials. In a late stage of our investigations, we found that the etching strategy is similar to^23^.

Etching of SiC involves a two-step process. First, oxidation of SiC follows two possible reaction pathways for the oxidation^24^:

1 $$\text{SiC} + 4\text{H}_{2} \text{O} + 8h^{ + } \to \text{SiO}_{2} + \text{CO}_{2} \uparrow + 8\text{H}^{ + }$$

2 $$\text{SiC} + 2\text{H}_{2}\text{O} + 4h^{ + } \to \text{SiO} + \text{CO} \uparrow + 4\text{H}^{ + } .$$

According to current knowledge, both oxidation pathways take place simultaneously, while the applied voltage determines the ratio of both^25^. Experimentally it is indeed reported that the dissolution valence, i.e. the number of charge carriers required to etch one formula unit of SiC is between 6 and 6.9^18,24^. In a second step, the resulting reaction products $\text{SiO}_\text{x}$ are removed by the electrolyte, that is potassium hydroxide (KOH). The dissolution of $\text{SiO}_{2}$ involves the adsorption of water with the formation of hydrated silica ($\text{SiO}_{2} + 2\text{H}_{2} \text{O} \to \text{Si}\left( {\text{OH}} \right)_{4}$) and an attack by hydroxyl ions to form a soluble silicate ($\text{Si}\left( \text{OH} \right)_{4} + 2\text{OH}^{ - } \to \left[ {\text{Si}\left( {\text{OH}} \right)_{2} \text{O}_{2} } \right]^{2 - } + 2\text{H}_{2} \text{O}$)^26,27^.

**Figure 2:**
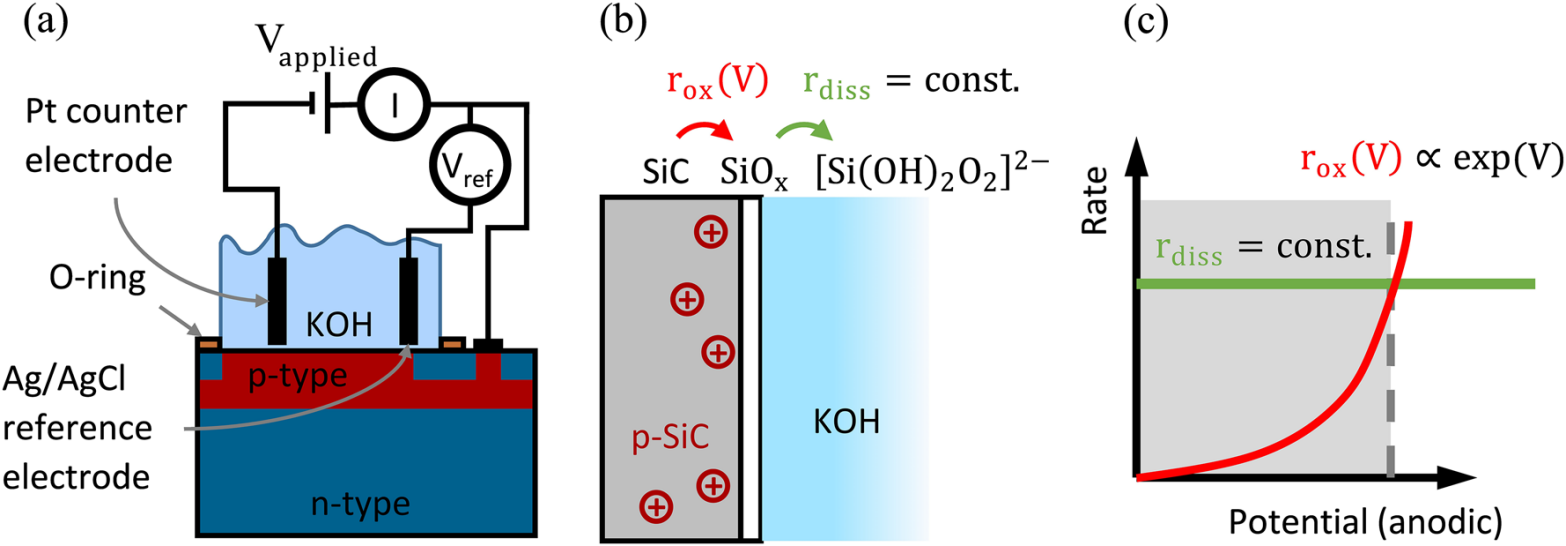
Electrochemical oxidation reactions. (**a**) Electrochemical etching setup. The current (I) flows via the p-SiC layer to the counter electrode. (**b**) Dissolution of $\text{SiC}$, forming $\text{SiO}$ and $\text{SiO}_{2}$ as intermediates, subsequently forming soluble silicates. (**c**) Oxidation ($\text{r}_{{{\text{ox}}}}$) and dissolution ($\text{r}_{\text{diss}}$) rates as a function of applied voltage. The grey area indicates the voltage range suitable for steady-state etching.

In steady state, the oxidation of SiC and the subsequent dissolution of $\text{SiO}_\text{x}$ occur with the same rate, which is our desired regime of operation, see Fig. 2. Otherwise, an overshooting of the $\text{SiO}_\text{2}$-formation would passivate the surface and block the electrochemical process. This would result in an oscillatory behavior at higher rates, which we avoided by sticking to low reaction rates^28,29^.

This electrochemical reaction gives a handle to remove specific volumina selectively. p-doped, i.e. $h^{ + }$-rich regions can efficiently be etched as opposed to n-doped regions where, due to the lack of $h^{ + }$, etching is completely suppressed.

**Figure 3:**
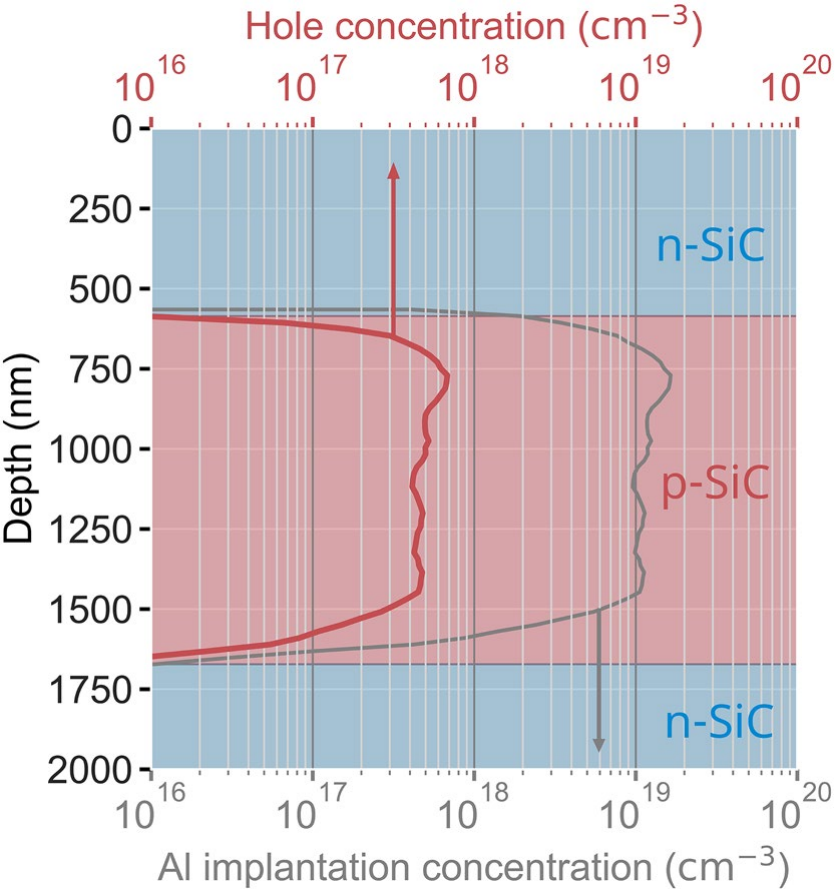
Dopant-defined layers for electrochemical etching. By suitable implantation profiles for Aluminum and Nitrogen, a sharply defined p-SiC layer with an excess of positive charge carriers (holes) is defined. The hole concentration is calculated, utilizing the charge neutrality equation, assuming a compensation ratio of 0.35^32^, and a doping concentration dependant ionization energy ($E_{{{\text{ion}}}} \left( {N_{{{\text{Al}}}} } \right) = 210 {\text{meV}} - 3 \cdot 10^{ - 8} {\text{eVcm}} \cdot N_{{{\text{Al}}}}^{1/3}$)^33,34^.

We start with a 4°-off-axis n-type wafer with an epilayer (nitrogen-doped 10^16^ cm^−3^). The required doping profiles can be defined by ion implantation, which is described in detail in the SI, detailed data can be found in^30^. The hole concentration profile is shown in Fig. 3, where Aluminum-implantation creates a box-like p-type profile in a depth from 550 nm to 1.6 µm. At its flank, the hole concentration drops by more than ten orders of magnitude within 50 nm, which gives hope that the etch-stop is defined within atomic precision. In order to ensure reliable n-doping of the top layer, a counter implantation with Nitrogen is performed. Subsequent annealing to 1700°C for 30 min in 900 mbar Argon-atmosphere re-establishes the crystalline lattice (locally, a carbon cap stabilizes the surface^31^). Note that dopant diffusion is essentially absent in the rigid SiC-lattice.

For the geometries in this manuscript, we used only vertical implantation profiles. The methodology can be readily extended to more complex 3D structures, when in addition, lateral patterning of the implantation is achieved, for example with robust metallic masks.

**Figure 4:**
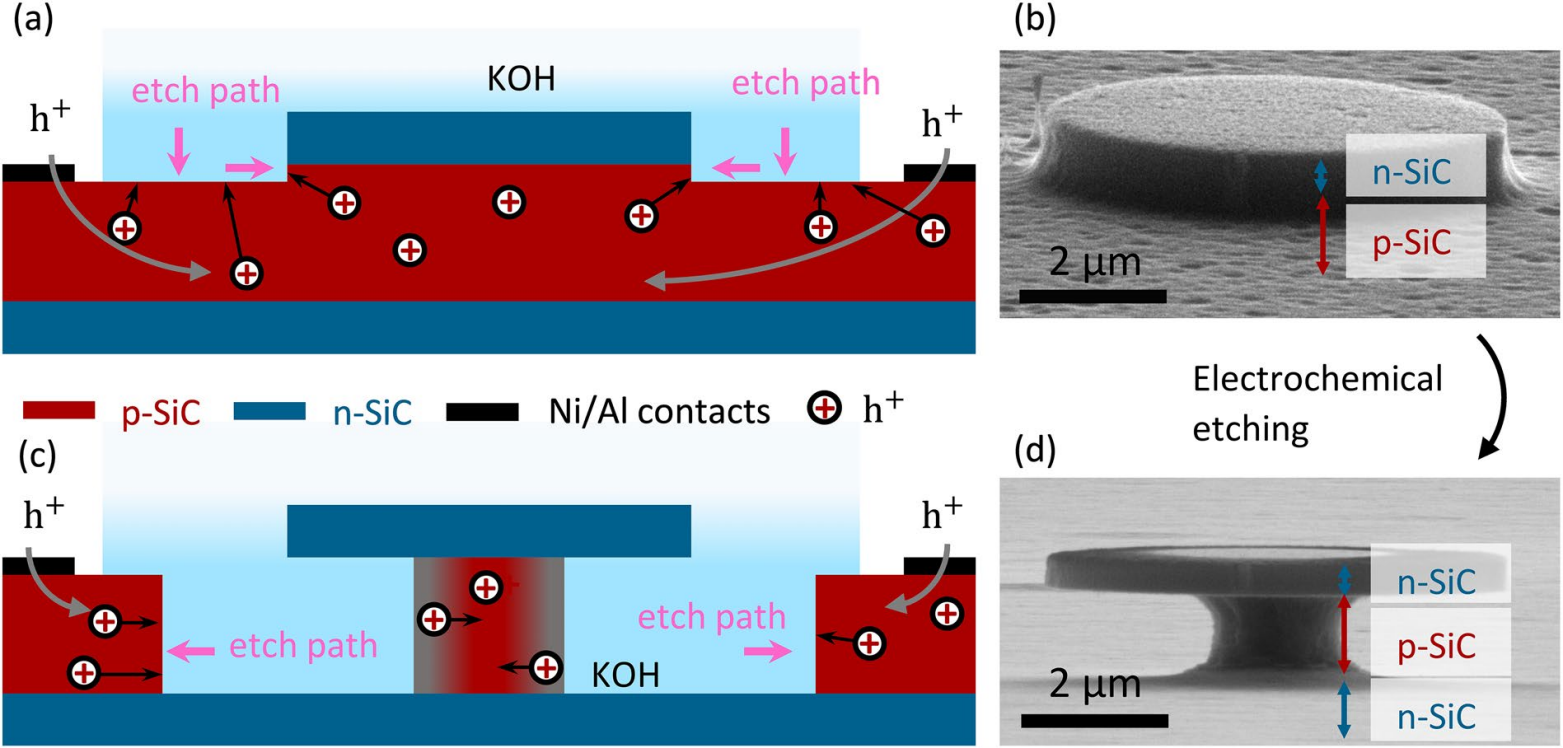
Electrochemical etching using dopant-defined layers. (**a**,**b**) Before ECE, areas to be removed are defined by lithography and gas etching slightly into the p-layer. Positive charge carriers (*h*^+^), required for ECE, are supplied via Ni/Al ohmic contacts. Applying anodic voltages results in (energy) band bending of the semiconductor, holes accumulate at the p-SiC/KOH interface and promote etching. (**c, d**) ECE removes the p-SiC layer. The etching is stopped vertically by the n-SiC layer; the lateral etching is stopped as soon as the remaining p-island is electrically unconnected. Without any applied potential, the p-SiC/KOH interface depletes of holes (due to band bending, grey area). (**b**,**d**) SEM micrographs, scale-bar: 2 µm.

But also with laterally homogeneous doping profiles, 3D structures can be defined. For this purpose, we pattern resist masks which define a top window (electron beam lithography or similar). A projective etching of the n-type layer is performed by standard RIE / ICP-RIE techniques, such that the p-SiC layer is slightly etched. Now, the ECE is performed, which isotropically removes the p-SiC layer, see Fig. 4. A typical lateral etch velocity is 2 µm/h. Care has to be taken that during this process an uninterrupted current path through the p-type layer has to be maintained. If however, p-type areas are disconnected from the current pathway during the etching, the dissolution stops for this island. While this may occur unintendedly, this property can also be exploited for the positive (see e.g. self-limited support columns for disk-shape optical resonators in Fig. 4). In any case, maintaining intact current pathways throughout the etching process has the rank of a design principle.

Figure 1 displays a cantilever-like structure after ECE. We report one complication that arises after the ECE. Underneath the top n-type layer, in barely accessible regions, often an undesired porous p-type structure remains (goat beard), see SI. It reminds the formation of porous SiC in KOH^35^. It can reliably be removed by two simple techniques: either a subsequent isotropic dry etch with CF_4_ at 190 mTorr that is suited for well-accessible devices like cantilevers, see SI. Alternatively, high temperature annealing beyond 1000°C in 900 mbar Argon atmosphere removes this layer even in hardly accessible regions. It can be suspected that thermal oxidation has a similar effect^36,37^.

**Figure 5:**
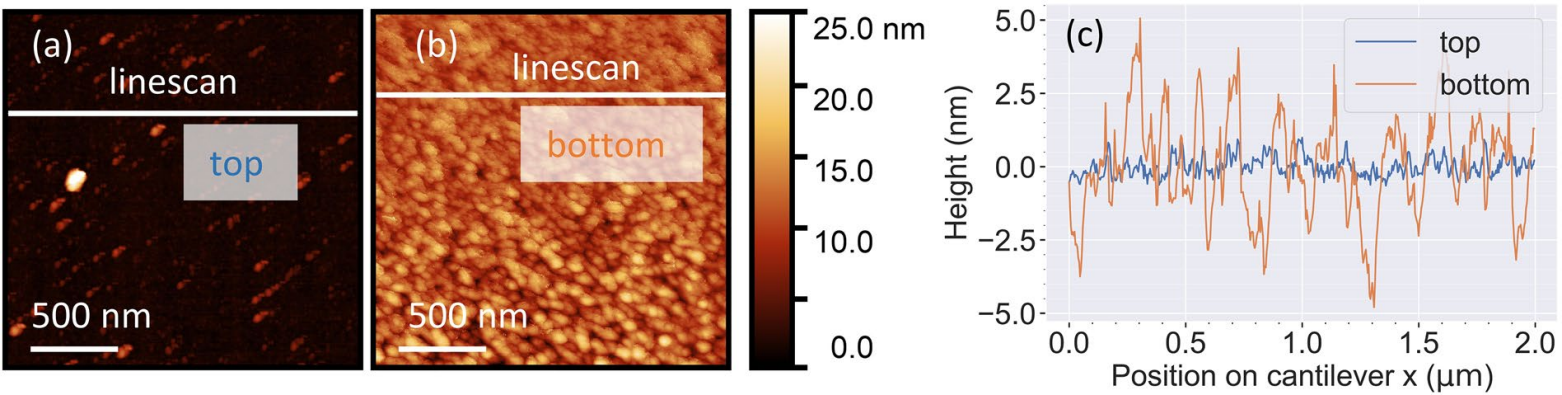
Surface characterization after ECE. AFM measurements on a cantilever (top and bottom surfaces). Statistical evaluation yields roughnesses of rms_top_ = 1.46 nm and rms_bottom_ = 2.48 nm. The scale-bar refers to 500 nm. (**b**) Height profiles referring to the lines indicated in (**a**).

An obvious quality criterion for optical or mechanical devices is the surface roughness. In our devices, the ECE process leaves the etched surface quite smooth. The top surface of the n-SiC layer is essentially unchanged (in our experiments, rms_top_ = 1.46 nm, see Fig. 5a). For characterizing the bottom layer, we removed a single clamped cantilever with scotch tape and studied its surface with the AFM. The result is shown in Fig. 5b, it yields rms_bottom_ = 2.48 nm. Hence, both the unetched top and the freshly etched bottom surfaces have both low surface roughness.

**Figure 6:**
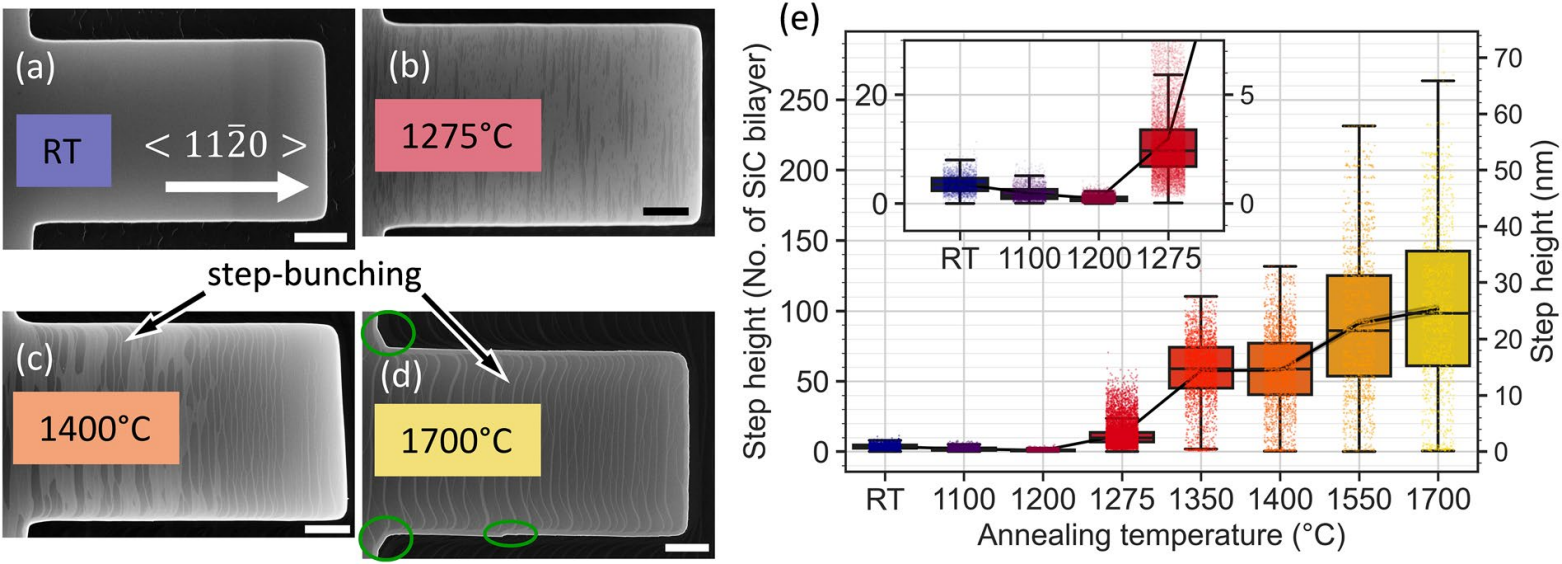
Cantilever shape evolution during high-temperature annealing. (**a**–**d**) SEM micrographs of the very same cantilever after step-wise annealing at selected temperatures. Faceted reconfigurations are highlighted in green. Scale-bar: 2 µm. (**e**) AFM line-scans were performed in the < 11$\overline{2}$0 > direction. The plot shows the analysis of the step height of this annealed cantilever as a function of annealing temperature. The SiC step height significantly increases for annealing temperatures above 1275°C. The step height is additionally given in units of SiC bilayer,  0.25 nm. The insert is a zoom emphasizing the low-temperature range.

Finally, we address the question, how the presented 3D fabrication technique is compliant with further processing. Underetched devices were spin-coated with PMMA and nLof resist materials without being damaged. They also survived lift-off processes, rinsing and drying without special precautions. Remarkably, the devices are also robust with respect to high-temperature steps. In SiC, relevant spin-carrying color centers are created, converted and finally annealed out in a temperature range from 400°C to 1400°C^12^. The native oxide layer sublimes at temperatures above 800°C in UHV^38^. Epitaxial graphene fabrication in n-type 4H-SiC is performed at above 1500°C^39^. Unintentional implantation damage anneals out at 1600°C to 1700°C. Hence, we explored this entire temperature range with completely processed devices like cantilever structures or membranes. They were exposed to high temperature steps in Ar atmosphere (900 mbar) for 30 min and subsequently investigated with SEM (see Fig. 6a–d) and AFM (Fig. 6e). The shape of the cantilevers is maintained at least up to 1550°C. Beyond this temperature, as can be seen in Fig. 6d a visible re-arrangement occurs. It is most obvious at the lower edge, where a discontinuity has been created. Also in the lower and upper left corners, an additional faceted transition is formed, following crystalline directions. Remarkably, below 1550°C, our cantilevers provide an excellent shape stability. An analysis of the upper surface profile shows very little effect up to 1200°C. In the temperature range of 1275°C to 1350°C, pronounced terraces are formed and step bunching occurs (4° miscut) with typical step heights of the order of 10 nm, beyond 1550°C approaching towards 25 nm.

From the contrast in the SEM micrographs, the characteristic pattern of the initiation of graphene growth becomes apparent, which forms a homogenous coverage when annealing the sample at 1700°C^11^. This is more than an interesting detail: epitaxial graphene provides an atomically smooth surface termination that is inert as long as oxygen plasma is avoided.

In conclusion, we present a versatile electrochemical fabrication route for generating high-quality monolithic 3D devices in SiC. The shape of the 3D structure is defined by doping profiles. The surface quality can be enhanced by high temperature annealing. While in this study, we limited ourselves to homogenous p-type layers and homogeneous n-type top layers, much more refined 3D shaping is possible. Such 3D devices, monolithically carved out of monocrystalline SiC wafers, pave the way to implement mechanical and optical monolithic devices with excellent surface properties on the SiC platform. Together with the already available electrical semiconductor functionalities, graphene electronics and spin-physics in SiC a rich toolbox can be established, unifying quantum and classical technologies on the very same chip (Supplementary Information).

### Supplementary Information

Supplementary Figures.

## Data Availability

The data that support the findings of this study is available in an open-access repository: https://doi.org/10.22000/1722.
