# Supplementary material for: Electrochemical etching strategy for shaping monolithic 3D structures from 4H-SiC wafers
Source: Sci Rep. 2023 Nov 4;13:19086. doi: 10.1038/s41598-023-46110-2 (PMC10625639; doi:10.1038/s41598-023-46110-2)
Supplement: Supplementary file 1 — Supplementary Figures. [file 41598_2023_46110_MOESM1_ESM.pdf]

Supplementary Information: Electrochemical etching strategy for shaping monolithic 3D structures from 4H-SiC wafers

*André Hochreiter, Fabian Groß, Morris-Niklas Möller, Michael Krieger, Heiko B. Weber**

*heiko.weber@fau.de

Lehrstuhl für Angewandte Physik, Department of Physics, Friedrich-Alexander-Universität Erlangen-Nürnberg (FAU), 91058 Erlangen, Germany

<u>Implantation:</u>

- A 50 nm Al scattering layer is deposited before implantation.
- The simulated implantation profile computed with TRIM [1] software is shown in **Figure S1**. The dark grey shaded area represents the Al scattering layer (the SiC surface is at the depth of 0 nm); a detailed collection of the implantation profiles used for our experiment is given in [2] together with a script to reproduce **Figure S1** as well as **Figure 2** of the main manuscript.
- The sample is kept at room temperature during the implantation. The implantation is conducted with our two in-house ion implanters covering energy ranges from 10 keV up to 2 MeV.
- The ion source was a hot cathode source for Nitrogen and a sputter source for Aluminum.

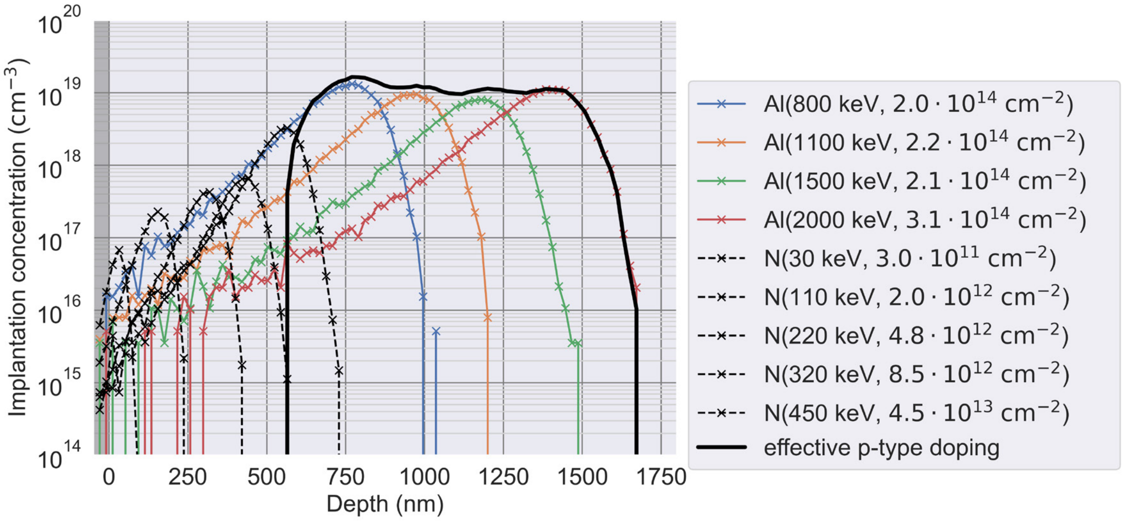

Figure S1: TRIM implantation profile. Grey area indicates the 50 nm Al scattering layer. Aluminium (Al) is used for p-doping. A counter-implantation of Nitrogen (N) is conducted to re-establish a top n-type SiC layer. The solid black line represents the effective p-type doping concentration, i.e. the sum of all Al implantation profiles minus the sum of the N counter-implantation profiles.

<u>Post-implantation annealing:</u>

- A post-implantation annealing for 30 minutes at 1700°C (900 mbar Ar) with a carbon-cap (C-cap) is needed in order to reduce implantation damage and activate the dopants [3, 4].

Electrochemical etching:

- A 1M KOH (1 mol/liter) solution is used.
- Electrochemical etching is conducted at room temperature without stirring.
- The sample is etched at an applied voltage of 1.65 V for 22 h resulting in an undercut of 31 µm (etch rate = 1.4 µm/h). The sample contains more than 50 different cantilever structures.
- In **Figure S2**, the time evolution of the etching current and the reference voltage are displayed, see also [2] for details.

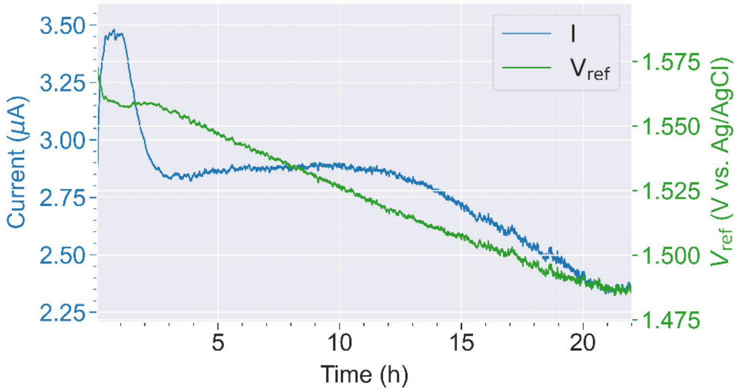

Figure S2: Electrochemical etching characteristics for the fabrication of free-standing cantilevers. Current and reference voltage over time.; etching duration of 22 h at 1.65 V.

High-temperature annealing experiments:

- Annealing is performed in a vertical cold-wall reactor comprising a double-walled, water-cooled quartz tube and a graphite susceptor in Ar flow. No bake out in vacuum was performed. The sample was annealed under 900 mbar Ar atmosphere for 30 minutes and subsequently cooled down for 1 h.
- Graphene growth takes place via sublimation of Si from the surface ($SiC(s) \rightarrow Si(g) + C(s)$), with C atoms diffusion and nucleation into graphene islands [5, 6]. As Si and C atoms are bonded more weakly on the step edges, Si desorption and graphene growth is faster on the step edges as on the terraces. Different decomposition velocities of terraces in 4H-SiC result in step bunching, at temperatures above 1200°C [6]. Here we utilize a Silicon Carbide container as supply of a background pressure of silicon vapor to prevent further loss of Si from the sample (often referred to as "sublimation sandwich" technique), resulting in higher quality graphene growth [5].

Porous p-SiC removal:

- After electrochemical etching, an undesired porous p-type structure often remains (goat beard), see **Figure S3** (a) and (b).
- Using a homogenous Ni hard mask as etch mask (50 nm homogeneously sputtered on the whole sample), this porous p-SiC can be removed by an isotropic dry etch in a RIE: 190 mTorr, $CF_4 = 20.4\ \text{sccm}, N_2 = 10.6\ \text{sccm}, O_2 = 12.2\ \text{sccm}, 300\ \text{W}$.
- While the homogeneous Ni mask protects the top surface and the sidewalls, the thin porous p-SiC is successfully removed, see **Figure S3** (c) and (b).

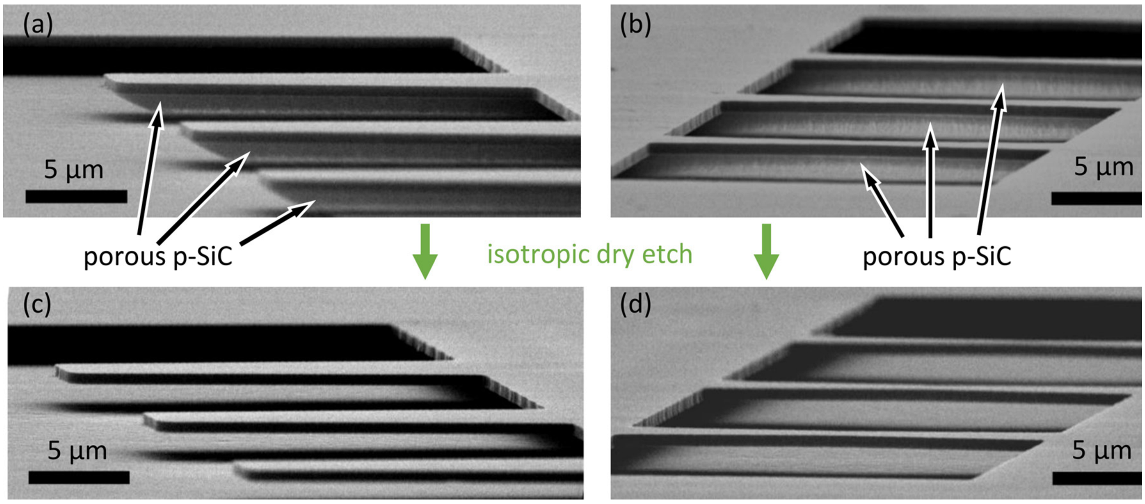

Figure S3: Porous p-SiC after ECE. (a) / (b) Cantilever directly after ECE with remaining porous p-SiC. (c) / (d) Cantilever after removal of porous p-SiC by isotropic dry etching (RIE, 190 mTorr, $CF_4 = 20.4\ \text{sccm}, N_2 = 10.6\ \text{sccm}, O_2 = 12.2\ \text{sccm}, 300\ \text{W}$, using a Ni hard mask). Scale-bar of SEM micrographs: 5 µm.

REFERENCES

[1] J. F. Ziegler and J. P. Biersack, „The stopping and range of ions in matter," in *Treatise on Heavy-Ion Science: Volume 6: Astrophysics, Chemistry, and Condensed Matter*, Springer, 1985, p. 93–129.

[2] A. Hochreiter and H. B. Weber, *Research data for the manuscript: Electrochemical etching strategy for shaping monolithic 3D structures from 4H-SiC wafers,* DOI: 10.22000/1722.

[3] J. Weiße, M. Hauck, M. Krieger, A. J. Bauer and T. Erlbacher, „Aluminum acceptor activation and charge compensation in implanted p-type 4H-SiC," *AIP Advances,* Bd. 9, p. 055308, 2019.

[4] M. Rambach, A. J. Bauer and H. Ryssel, „Electrical and topographical characterization of aluminum implanted layers in 4H silicon carbide," *physica status solidi (b),* Bd. 245, p. 1315–1326, 2008.

[5] B. K. Daas, S. U. Omar, S. Shetu, K. M. Daniels, S. Ma, T. S. Sudarshan and M. V. S. Chandrashekhar, „Comparison of epitaxial graphene growth on polar and nonpolar 6H-SiC faces: on the growth of multilayer films," *Crystal growth & design,* Bd. 12, p. 3379–3387, 2012.

[6] G. R. Yazdi, T. Iakimov and R. Yakimova, „Epitaxial graphene on SiC: a review of growth and characterization," *Crystals,* Bd. 6, p. 53, 2016.

[7] J. Weisse, M. Hauck, T. Sledziewski, M. Tschiesche, M. Krieger, A. J. Bauer, H. Mitlehner, L. Frey and T. Erlbacher, „Analysis of compensation effects in aluminum-implanted 4H-SiC devices," in *Materials Science Forum*, 2018.
